# The role of acyl cycling in endogenous G protein localization

**DOI:** 10.1016/j.jbc.2025.111045

**Published:** 2025-12-12

**Authors:** Wonjo Jang, Kanishka Senarath, Sumin Lu, Gonzalo P. Solis, Vladimir L. Katanaev, Nevin A. Lambert

**Affiliations:** 1 Department of Pharmacology and Toxicology, Medical College of Georgia, Augusta University, Augusta, Georgia, USA; 2 Translational Research Center in Oncohaematology, Department of Cell Physiology and Metabolism, Faculty of Medicine, University of Geneva, Geneva, Switzerland; 3 Translational Oncology Research Center, Qatar Biomedical Research Institute, Hamad Bin Khalifa University, Doha, Qatar

**Keywords:** G proteins, heterotrimeric G proteins, HRas, palmitoylation, depalmitoylation, CRISPR, BRET

## Abstract

Monomeric and heterotrimeric G proteins associate with membranes in part due to S-acylation, most often the reversible addition of palmitate (C16). An ongoing cycle of deacylation, shuttling through the cytosol and reacylation, is known to be important for the steady-state enrichment of monomeric G proteins at the plasma membrane, but it is not known whether heterotrimeric G proteins are similarly regulated. Here we study how deacylation and reacylation affect the subcellular distribution of endogenous heterotrimeric G proteins and compare this to endogenous HRas. Pharmacological inhibition of palmitoyl transferase (PAT) enzymes had almost no effect on the subcellular distribution of endogenous heterotrimers but redistributed endogenous HRas from the plasma membrane to intracellular membranes. Similarly, redirecting PAT enzymes to the outer nuclear membrane trapped a large fraction of endogenous HRas in this compartment, whereas endogenous heterotrimers were only minimally affected. Our results suggest that acyl cycling is not important for the steady-state subcellular distribution of heterotrimeric G proteins, and that heterotrimers do not have ready access to intracellular membrane compartments through the cytosol.

Heterotrimeric G proteins transduce signals detected by G protein-coupled receptors (GPCRs) to regulate a wide variety of important physiological processes. While many important signaling events take place at the plasma membrane, it is now recognized that GPCRs also activate G proteins on several intracellular organelles (1, 2, 3, 4). Therefore, it is important to understand how heterotrimeric G proteins are distributed across subcellular compartments and how this distribution is maintained and regulated. Heterotrimers consist of Gα subunits and Gβγ dimers, both of which are attached to cellular membranes by posttranslational lipid modifications; Gα subunits are myristoylated and/or S-acylated (5, 6, 7, 8, 9), and Gγ subunits are prenylated and carboxymethylated (10). Similarly, many monomeric G proteins of the Ras family are anchored to membranes by a combination of S-acylation and prenylation, where they transduce signals detected by growth factor receptors (11). The combined influence of multiple lipid modifications confers stable association with the plasma membrane (12), the primary (but not exclusive) site of action of both types of G protein.

Prenylation and N-myristoylation are stable modifications, whereas S-acylation (most often with palmitate) is reversible. Depalmitoylation allows Ras proteins such as NRas and HRas to dissociate from the plasma membrane, transit the cytosol, and reversibly associate with intracellular membranes (13, 14). Repalmitoylation by Asp-His-His-Cys (DHHC) motif-containing palmitoyl transferase (PAT) enzymes (15, 16) at the Golgi apparatus traps NRas and HRas on Golgi membranes, and vesicular transport returns these proteins to the plasma membrane (Fig. 1*A*). This deacylation–reacylation cycle is widely accepted as an explanation for how Ras proteins avoid randomization on cellular membranes and remain concentrated on the plasma membrane (17). In contrast, the role of acyl cycling in the subcellular localization of heterotrimeric G proteins is not well understood. The palmitate chains attached to Gα subunits are known to turn over (18, 19), and it has been suggested that a deacylation–reacylation cycle regulates heterotrimer localization in much the same way that acyl cycling regulates Ras proteins (17). It has been further suggested that steady-state depalmitoylation occurs at a rate sufficient to allow depalmitoylated heterotrimers to frequently “shuttle” through the cytosol and sample intracellular compartments (20, 21). However, many questions about acyl cycling and heterotrimer trafficking have not been addressed. One important unanswered question is the extent to which this mechanism applies to endogenous G proteins, since most studies of G protein trafficking have relied primarily on overexpressed proteins. The use of overexpressed subunits is particularly problematic for heterotrimeric G proteins because Gα and Gβγ do not traffic appropriately when expressed alone (22, 23, 24), and aberrant localization is sometimes observed even when all three subunits (Gα, Gβ, and Gγ) are expressed together. Here we study the role of acyl cycling in the subcellular localization of endogenous heterotrimeric G proteins using pharmacological and genetic approaches and endogenous HRas as a positive control. We find that acyl cycling plays at most a minor role in the subcellular distribution of endogenous heterotrimeric G proteins.

**Figure 1:**
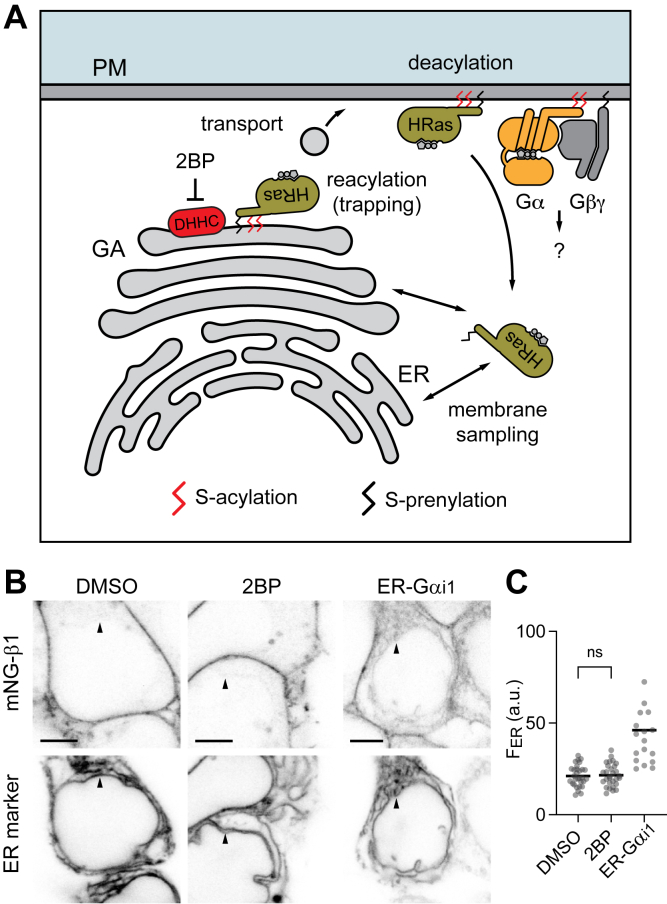
**Blocking PAT activity does not redistribute endogenous mNG-β1.***A*, deacylation (depalmitoylation) at the plasma membrane (PM) allows HRas to sample intracellular membranes including the endoplasmic reticulum (ER). Reacylation by DHHC PAT enzymes at the Golgi apparatus (GA) traps HRas for transport back to the PM. Heterotrimeric (Gα + Gβγ) G proteins have similar lipid modifications and could traffic *via* a similar mechanism. *B*, confocal images of mNG-β1 cells expressing the ER marker mRuby2-PTP1b after treatment with vehicle (DMSO) or 2-bromopalmitate (2BP; 50 μM) for 6 h, or after coexpression of an ER-localized Gα subunit (ER-Gαi1); scale bars 5 μm. *C*, mNG-β1 fluorescence at the ER (F_ER_) was not significantly different after treatment with DMSO (*n* = 30 cells on 3 coverslips) or 2BP (*n* = 29 cells on 3 coverslips; *p* = 0.98, ordinary one-way ANOVA with Dunnett’s multiple comparisons); redistribution of mNG-β1 to the ER was obvious after coexpression of ER-Gαi1 (*n* = 17 cells on 3 coverslips).

## Results

### Blocking PAT enzymes redistributes endogenous HRas but not heterotrimers

To study the role of acyl cycling in the distribution of endogenous heterotrimeric G proteins we first performed confocal imaging studies using gene-edited HEK 293 cells with Gβ1 subunits labeled with complementing fragments of the green fluorescent protein mNeongreen2 (mNG-β1 cells). Many previous studies have shown that monomeric Ras proteins redistribute from the plasma membrane to intracellular membranes when DHHC PAT enzymes are blocked with 2-bromopalmitate (2BP; Fig. 1*A*) (13, 14, 25). To look for a similar redistribution of heterotrimeric G proteins, we measured mNG-β1 fluorescence in intracellular regions of interest defined by a red marker of the endoplasmic reticulum (ER); this organelle has a large surface area and is sampled by translocating Gα subunits and Gβγ dimers (26, 27). As we have reported previously (27), mNG-β1 was essentially undetectable on the ER of vehicle-treated cells, and treatment with 2BP did not increase mNG-β1 fluorescence associated with this compartment (Fig. 1*B*). Since free Gβγ dimers are not stably associated with the plasma membrane (28) we expected that mNG-β1 subunits would follow Gα subunits that redistributed to the ER. To test this expectation, we coexpressed Gα subunits bearing an ER-targeting motif (ER-Gαi1) and found that this led to a marked increase in mNG-β1 fluorescence at the ER (Fig. 1*B*). These findings suggested that acyl cycling does not affect the subcellular distribution of heterotrimeric G proteins to the extent observed previously for small G proteins and further indicated that the subcellular localization of endogenous Gβ subunits is dictated by the localization of Gα subunits.

**Figure 2:**
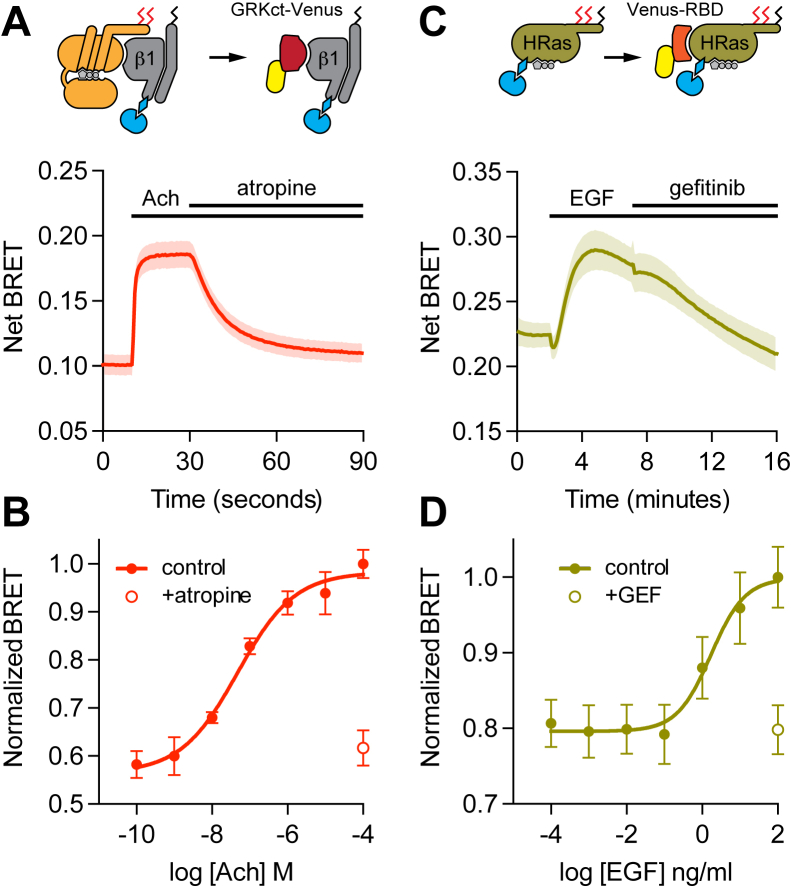
**Functional validation of HiBit-β1 and HiBit-HRas cells.***A*, HiBit-β1 cells expressing LgBit and M4 muscarinic acetylcholine (Ach) receptors were stimulated with Ach (100 μM) followed by the antagonist atropine (100 μM). Activation of heterotrimers containing HiBit-β1 was detected using the free Gβγ sensor memGRKct-Venus. Mean ± 95% CI of 36 replicates from 3 independent experiments. *B*, steady-state concentration-dependence of heterotrimer activation as in *A*. Mean ± S.D. of 9 replicates from 3 independent experiments. *C*, HiBit-HRas cells expressing LgBit were stimulated with epidermal growth factor (EGF; 100 ng ml^-1^) followed by the antagonist gefitinib (GEF; 1 μM). Activation of HiBit-HRas was detected using the Ras binding domain of Raf1 fused to Venus (Venus-RBD). Mean ± 95% CI of 16 replicates from 4 independent experiments. *D*, steady-state concentration-dependence of HiBit-HRas activation as in *C*. Mean ± S.D. of 30 replicates from 5 independent experiments.

**Figure 3:**
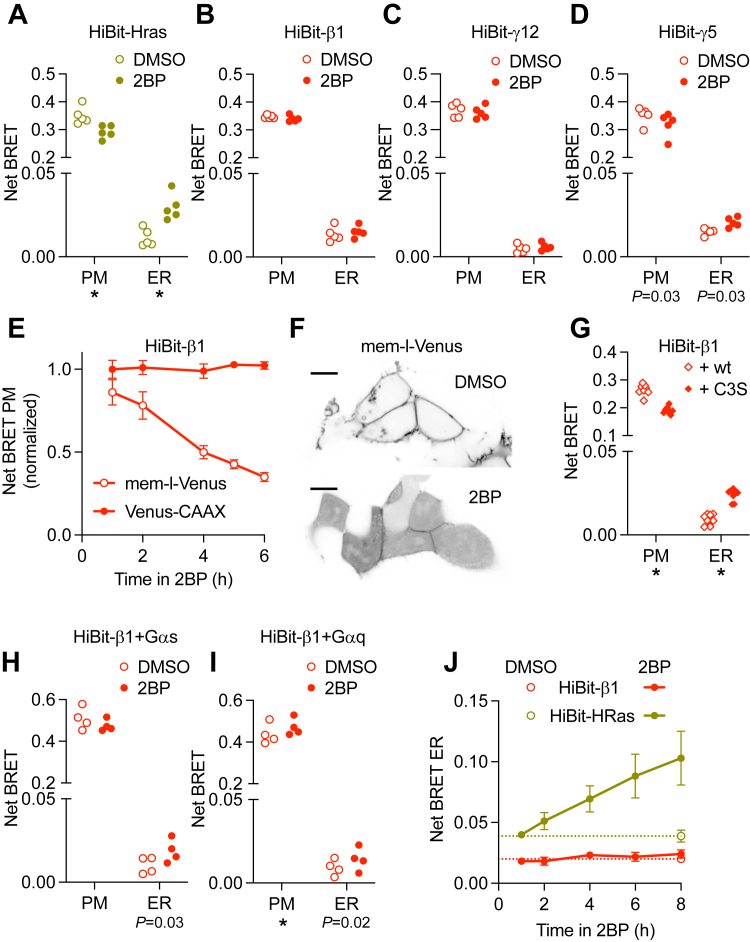
**Blocking PAT activity redistributes HiBit-HRas but not HiBit-β1.***A*, bystander BRET between HiBit-HRas and markers of the plasma membrane (Venus-CAAX; PM) or endoplasmic reticulum (ER; Venus-PTP1b) after 6-h treatment with vehicle (DMSO) or 2-bromopalmitate (2BP; 50 μM); *n* = 5. *B–D*, similar to A with HiBit-β1, HiBit-γ12 or HiBit-γ5 cells; *n* = 5. *E*, bystander BRET between HiBit-β1 and Venus-CAAX or mem-l-Venus during treatment with 50 μM 2BP (normalized to DMSO treatment for 6 h); mean ± SD of 4 independent experiments. *F*, representative confocal images of HiBit-β1 cells expressing mem-l-Venus after 6-h treatment with DMSO or 2BP; scale bars 10 μm. *G*, bystander BRET between HiBit-β1 and PM or ER markers with overexpression of wild-type (wt) Gαi1 or Gαi1 C3S; *n* = 8. *H* and *I*, bystander BRET between HiBit-β1 and PM or ER markers after 6-h treatment with DMSO or 2BP with overexpression of Gα_s_ (*H*) or Gα_q_ (*I*); *n* = 4. *J*, bystander BRET between HiBit-HRas or HiBit-β1 and the ER during treatment with DMSO or 2BP for 8 h; mean ± SD of 5 independent experiments. For *A-D* and *G-I*, *p* < 0.01 is indicated with an asterisk∗ and *p* values between 0.01 and 0.05 are shown, paired *t* test.

These results prompted us to perform similar studies using bystander bioluminescence resonance energy transfer (BRET), as this method has high sensitivity and can be performed on large populations of cells (29). Accordingly, we generated gene-edited HEK 293 cell lines with the small peptide HiBit added to the N terminus of Gβ1 subunits or the N terminus of HRas. SDS-PAGE analysis of HiBit-HRas cells revealed a single HiBit-tagged protein with the expected molecular weight, and bystander BRET experiments with coexpressed LgBit revealed subcellular localization consistent with endogenous HRas (Fig. S1) (30); similar results were reported previously for HiBit-β1 cells (27). We then verified that both HiBit-β1 and HiBit-HRas retained functionality using BRET assays that detect free HiBit-βγ dimers after activation of a G protein coupled receptor (GPCR; Fig. 2, *A* and *B*) and GTP-bound HiBit-HRas after activation of endogenous growth factor receptors (Fig. 2, *C* and *D*). We then monitored the subcellular distribution of endogenous HiBit-β1 and HiBit-HRas by coexpressing BRET acceptors targeted to the cytosol-facing surfaces of the plasma membrane (Venus-CAAX) or endoplasmic reticulum (Venus-PTP1b). Bystander BRET signals are generated only when BRET donors and acceptors are present on the same membrane surface, and changes in bystander BRET accurately reflect changes in membrane protein abundance. Endogenous HiBit-HRas and HiBit-β1 showed high bystander BRET signals at the plasma membrane and very low signals at the ER, consistent with our imaging results and previous studies of these proteins (Fig. 3, *A* and *B*). Treatment with 2BP for 6 h resulted in a significant (∼20%) decrease in HiBit-HRas abundance at the plasma membrane and a proportionally larger (>100%) increase in HiBit-HRas abundance at the ER (Fig. 3*A*), consistent with many previous studies of overexpressed HRas after treatment with 2BP. In contrast, 2BP had no detectable effect on HiBit-β1 abundance at either of these compartments (Fig. 3*B*). To corroborate this result, we performed similar experiments with edited cell lines with the HiBit peptide added to the N terminus of Gγ5 and Gγ12 subunits, the most abundant Gγ subunits expressed in HEK 293 cells (31). Similar to our results with HiBit-β1 cells, 2BP did not affect HiBit-γ12 distribution, whereas there was a small redistribution of HiBit-γ5 that approached statistical significance (defined as *p* < 0.01; paired *t* test; Fig. 3, *C* and *D*). To demonstrate that 2BP was effectively blocking PAT activity in HiBit-β1 cells, we performed similar experiments with a BRET acceptor targeted to the plasma membrane by palmitoylation (mem-link-Venus). Treatment with 2BP for 6 h decreased bystander BRET between HiBit-β1 and mem-link-Venus to <35% of the vehicle-treated control (Fig. 3*E*), presumably because deacylated mem-link-Venus left the plasma membrane (Fig. 3*F*). These bystander BRET results confirm that blocking acylation with 2BP redistributes endogenous HRas from the plasma membrane to intracellular compartments within a few hours and suggest that heterotrimeric G proteins do not undergo a similar redistribution.

Because these experiments monitored the subcellular location of Gβγ subunits we wanted to demonstrate that this method could detect changes in distribution that depended on the lipid anchors of Gα subunits. Accordingly, we overexpressed either wild-type (wt) Gαi1 subunits, which are palmitoylated at residue C3, or Gαi1 C3S mutants that cannot be modified at this residue. Overexpression of Gαi1 C3S redistributed HiBit-β1 away from the plasma membrane and onto the ER when compared to wt Gαi1 (Fig. 3*G*). This result confirmed that mislocalization of Gα results in mislocalization of Gβγ, implying that net loss of palmitoylation of endogenous Gα subunits would produce a similar change.

Gα_i/o_ family subunits are more abundant in HEK 293 cells than Gα_s_, Gα_q_, or Gα_12/13_ family subunits (31). Gα_i/o_ family subunits are anchored by both myristoylation and palmitoylation whereas the other Gα subtypes are only palmitoylated (10). Therefore, one possible explanation for our failure to detect effects of 2BP on G protein localization is the relative paucity of heterotrimers containing Gα subunits that depend entirely on palmitoylation. To test this idea, we overexpressed Gα_s_ and Gα_q_ subunits to increase the fraction of the heterotrimer pool containing these subunits. Under these conditions, we still failed to detect substantial 2BP-induced redistribution of HiBit-β1 (Fig. 3, *H* and *I*); there was a trend toward redistribution from the plasma membrane to the ER when Gα_s_ or Gα_q_ was overexpressed, but this did not reach statistical significance.

To assess the rate at which endogenous HRas is depalmitoylated in these cells, we monitored the time course of 2BP-induced redistribution of HiBit-HRas, focusing on the increase in bystander BRET signals at the ER. These signals increased in an approximately linear fashion for 8 h after the addition of 2BP and did not reach steady-state during this period (Fig. 3*J*), suggesting that depalmitoylation of HRas may be slower in HEK 293 cells than in other cell types (13). Once again, 2BP treatment failed to induce a significant redistribution of HiBit-β1 to the ER (Fig. 3*J*).

### Mislocalized PAT enzymes redistribute endogenous HRas and heterotrimers to different extents

**Figure 4:**
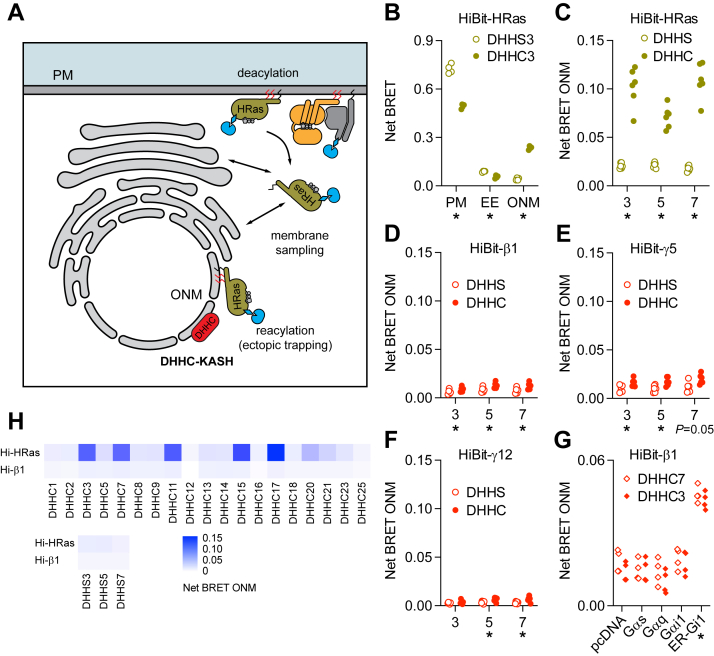
**Minimal redistribution of HiBit-β1 after expression of mislocalized PAT enzymes.***A*, directing DHHC enzymes to the outer nuclear membrane (ONM) with a KASH domain traps repalmitoylated substrates on this compartment. *B*, bystander BRET between HiBit-HRas and markers of the PM, ER, or ONM (Venus-KASH) 24 h after expression of catalytically-inactive DHHS3-KASH or catalytically-active DHHC3-KASH; *n* = 4. *C*, bystander BRET between HiBit-HRas and the ONM 24 h after expression of inactive (DHHS) or active DHHC3-, DHHC5- or DHHC7-KASH; *n* = 6. *D-F*, bystander BRET between HiBit-β1 (*D*), HiBit-γ5 (*E*) or HiBit-γ12 (*F*) and the ONM 24 h after expression of inactive (DHHS) or active DHHC3-, DHHC5- or DHHC7-KASH; *n* = 5. *G*, bystander BRET between HiBit-β1 and the ONM 24 h after expression of DHHC3- or DHHC7-KASH and empty vector (pcDNA), Gα_s_, Gα_q_, Gα_i1_, or ER-Gα_i1_; ∗*p* < 0.01, one-way ANOVA *versus* empty vector; *n* = 4. *H*, heatmap of net bystander BRET between HiBit-HRas and HiBit-β1 and the ONM after expression of a panel of mislocalized DHHC-KASH enzymes; each cell represents the mean of 3 independent experiments.

As an alternative approach to study how acyl cycling regulates the subcellular distribution of G proteins, we adapted the SwissKASH assay for bystander BRET (32). In this assay, DHHC PAT enzymes are fused to a KASH domain, leading to mislocalization of the enzymes on the outer nuclear membrane (ONM; Fig. 4*A*). Depalmitoylated proteins that can sample the ONM can then be repalmitoylated and directed to this compartment, provided they are substrates for the mislocalized PAT (DHHC-KASH). Negative controls are identical enzymes bearing C to S mutations that disable PAT catalytic activity (DHHS-KASH). We monitored mislocalization of G proteins by measuring bystander BRET between endogenous G proteins and a BRET acceptor directed to the ONM by the same KASH domain used to redirect PAT enzymes (Venus-KASH). We first validated this modified assay by overexpressing DHHC3-KASH in HiBit-HRas cells, which resulted in a significant decrease in HiBit-HRas abundance at the plasma membrane and a ∼5-fold increase in HiBit-HRas abundance at the ONM (Fig. 4*B*). Similar changes were observed after expression of DHHC5-KASH and DHHC7-KASH (Fig. 4*C*). These particular DHHC enzymes were chosen because they are thought to be the primary PATs that palmitoylate Gα subunits (21). Statistically significant redistribution of HiBit-β1 was observed 24 h after expression of DHHC-KASH enzymes, but the extent of redistribution was much smaller than what we observed with HiBit-HRas (<2-fold increase at the ONM; Fig. 4*D*). Similar modest but significant redistribution was seen for HiBit-γ5 and HiBit-γ12 (Fig. 4, *E* and *F*). Similar to our results with 2BP, overexpression of Gα_s_ or Gα_q_ did not alter the ability of DHHC-KASH enzymes to relocalize HiBit-β1 to the ONM (Fig. 4*G*). In contrast, overexpression of ER-Gαi1 (but not wild-type Gαi1) significantly increased HiBit-β1 localization to the ONM (Fig. 4*G*), presumably because the ONM and ER are physically continuous.

There are 23 different human DHHC PAT enzymes (33), and it is possible that Gα subunits might be better substrates for different DHHC enzymes than those tested above. Accordingly, we reexamined HiBit-HRas and HiBit-β1 localization after expression of a broader panel of 19 DHHC-KASH enzymes. Several additional DHHC-KASH enzymes were capable of redistributing endogenous HiBit-HRas to the ONM, most notably DHHC11-KASH, DHHC15-KASH and DHHC17-KASH (Fig. 4*H*). In contrast, none of the DHHC-KASH enzymes tested was able to induce comparable redistribution of endogenous HiBit-β1 (Fig. 4*H*).

### The effect of G protein activation on acyl cycle-dependent localization

**Figure 5:**
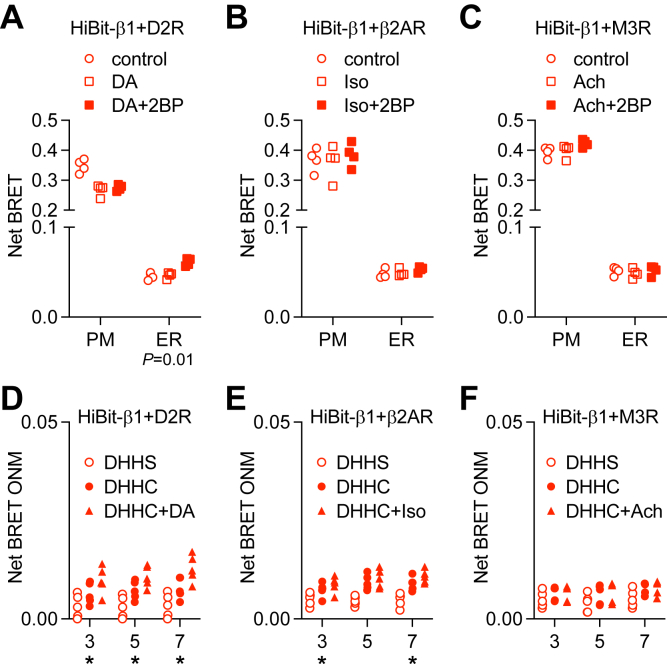
**Effect of activation on acylation-dependent HiBit-β1 localization.***A–C*, bystander BRET between HiBit-β1 and the PM and ER after 6-h activation of dopamine D2 receptors (D2R; *A*), β2 adrenoreceptors (β2AR; *B*), or M3 muscarinic acetylcholine receptors (M3R; *C*) with or without 6-h treatment with 2BP; ∗*p* < 0.01, one-way ANOVA, Tukey’s multiple comparisons, agonist alone *versus* agonist plus 2BP; *n* = 4. *D-F*, bystander BRET between HiBit-β1 and the ONM after 6-h activation of D2R (*D*), β2AR (*E*), or M3R (*F*) with or without overnight coexpression of DHHC3-, DHHC5-, or DHHC7-KASH; ∗*p* < 0.01, one-way ANOVA, DHHC alone *versus* DHHC plus agonist, Tukey’s multiple comparisons; *n* = 5.

It has been shown that G protein activation increases the rate at which Gα subunits are depalmitoylated (18, 19). Therefore, we asked how persistent G protein activation might affect acylation-dependent localization of heterotrimers. To do so, we expressed D2 dopamine receptors (D2R), β2 adrenergic receptors (β2AR) or M3 muscarinic acetylcholine receptors (M3R) to activate heterotrimers containing Gα_i/o_, Gα_s_ or Gα_q_ subunits, respectively. Together these three families account for the large majority of heterotrimers expressed in HEK 293 cells. We activated receptors for 6 h in the presence or absence of 2BP, then blocked receptor activation to allow the acute translocation of Gβγ to reverse. Control experiments verified that 2BP did not inhibit receptor-mediated G protein activation (Fig. S2). We then measured bystander BRET between HiBit-β1 and the PM and ER. Activation of D2R enabled a very small but significant 2BP-induced increase in HiBit-β1 abundance at the ER (Fig. 5*A*). Interestingly, activation of D2R alone led to a sustained loss of HiBit-β1 abundance at the PM, but this loss was not enhanced by 2BP. In contrast, activation of β2AR and M3R did not enable 2BP to promote redistribution of HiBit-β1 to the ER (Fig. 5, *B* and *C*).

We then performed similar experiments combining persistent activation of G proteins with overexpression of mislocalized DHHC-KASH enzymes. Similar to what we observed with 2BP, activation of D2R enhanced redistribution of HiBit-β1 to the ONM after expression of DHHC3-KASH, DHHC5-KASH, and DHHC7-KASH (Fig. 5*D*). Once again, although statistically significant the magnitude of HiBit-β1 redistribution under these conditions was quite small compared to what was observed with HiBit-HRas. A similar but even less pronounced enhancement was observed for activation of β2AR, and this was only significant for DHHC3-KASH and DHHC7-KASH (Fig. 5*E*). In contrast, activation of M3R did not enhance redistribution of HiBit-β1 after expression of any of the DHHC-KASH enzymes (Fig. 5*F*). Taken together, these results are consistent with previous studies showing increased depalmitoylation upon G protein activation (18, 19) but suggest that this increase has a modest impact on heterotrimer localization.

## Discussion

The main finding of these studies is that a deacylation–reacylation cycle is much less important for the steady-state subcellular distribution of endogenous heterotrimeric G proteins than endogenous HRas. Blocking PAT enzyme activity or overexpressing mislocalized PAT enzymes had negligible effects on heterotrimer distribution, whereas substantial redistribution was observed for HRas. Several factors could explain why acyl cycling is less important for heterotrimeric G proteins. One possible factor is differences in deacylation of endogenous heterotrimers and Ras proteins. Metabolic labeling experiments suggest that palmitate turnover half-lives are similar for Gα subunits and HRas (∼1.5–2 h) (19, 21, 34, 35), but such experiments cannot rule out a population of Gα that loses palmitate at a very slow rate. Our results with Gαi1 C3S (Fig. 3*G*) suggest that we should have detected significant redistribution of HiBit-β1 if a large fraction of endogenous Gα was depalmitoylated. Another possible contributing factor is that heterotrimers have stronger permanent anchoring mechanisms than monomeric G proteins, which could enable persistent association with the plasma membrane even after palmitate is lost. Most G protein γ subunits are geranylgeranylated (20 carbons), whereas Ras proteins are farnesylated (15 carbons) (10); geranylgeranylated proteins have a much higher affinity for membranes than farnesylated proteins (36). Gα subunits also have additional anchoring mechanisms; Gα_i/o_ subunits are myristoylated (7), and Gα_s_ and Gα_q_ both have polybasic surfaces that contribute to membrane association (37). Moreover, cGMP phosphodiesterase type 6 (PDE6δ) serves as an accessory protein to solubilize farnesylated Ras (38), whereas no similar protein is known to exist for mammalian geranylgeranylated Gγ subunits. UNC119 (a.k.a. RG4) has been shown to chaperone free myristoylated Gα_t_ subunits (39, 40), but expression of this protein is low outside of the retina. For these reasons, heterotrimers containing depalmitoylated Gα would be expected to dissociate from the plasma membrane much more slowly than depalmitoylated Ras proteins. Prolonged membrane association might allow depalmitoylated heterotrimers to be repalmitoylated in place at the plasma membrane and thus avoid redistribution through the cytosol. While most DHHC PAT enzymes are localized at the Golgi apparatus, several are localized at the plasma membrane (33), including DHHC5 and DHHC11. DHHC5 has been shown to palmitoylate Gα_q_, Gα_s_ and Gα_i_ subunits (21, 41) and DHHC11 has been shown to palmitoylate the N-terminal peptide of Gα_o_ (32). Therefore, either of these PATs could catalyze local repalmitoylation of Gα subunits at the plasma membrane. Because HRas is modified with two palmitates, it is possible that it too could lose one palmitate and then be repalmitoylated at the plasma membrane without entering the cytosol. Local repalmitoylation at the plasma membrane would be less likely for NRas, which is modified by a single palmitate; rapid dissociation of depalmitoylated NRas from the plasma membrane would increase the likelihood of repalmitoylation at the Golgi apparatus. Such a difference in primary sites of repalmitoylation could help explain why endogenous HRas is less abundant at the Golgi apparatus than endogenous NRas (30, 42). Altogether our present results are consistent with the idea that local palmitoylation is important for localization of peripheral membrane proteins at the plasma membrane (32, 43).

Our conclusion that endogenous heterotrimers do not regularly leave the plasma membrane contrasts with two previous studies that relied partly or entirely on overexpressed G protein subunits (20, 21). These studies found that overexpressed Gα subunits fused to fluorescent proteins were abundant at the Golgi apparatus and shuttled rapidly between this organelle and the plasma membrane. It is possible that overexpression promotes aberrant trafficking of G protein subunits, as we have found only a small population of endogenous heterotrimers on the medial Golgi apparatus of HEK 293 cells (27). This observation suggests that this compartment is not a major waypoint for the trafficking of endogenous heterotrimers after their initial synthesis. Parallel findings have been made for HRas, in that overexpressed HRas is more abundant at the Golgi apparatus than endogenous HRas (30). It is also worth noting that overexpressed HRas is more dramatically mislocalized after treatment with 2BP than what we observed with endogenous HRas (13, 14). An alternative explanation for differences between this study and prior studies of both types of G proteins is cell type-specific expression of the enzymes that catalyze depalmitoylation and repalmitoylation; acyl cycling could be more important for G protein trafficking in cells where depalmitoylation is rapid or repalmitoylation at the plasma membrane is slow (44). In either case, our results suggest that, at least in HEK 293 cells, heterotrimeric G proteins do not have ready access to intracellular compartments through the cytosol, and therefore heterotrimer distribution will be dominated by vesicle-mediated trafficking.

A limitation of our study is the reliance on labeled Gβ and Gγ subunits to infer localization of heterotrimers. Since free Gβγ dimers dissociate from the plasma membrane and sample all intracellular membranes (45) it is likely that the steady-state distribution of Gβγ that we observe accurately reflects the overall distribution of heterotrimers, as our data suggest. However, our assays do not report localization or movement of free Gα subunits. For example, it is known that a fraction of Gα_s_ leaves the plasma membrane when activated (46, 47), although the role of palmitoylation and depalmitoylation in Gα_s_ translocation to and from the plasma membrane has not been conclusively demonstrated (26, 48). Similarly, our experiments do not address the role of acyl cycling in the steady-state localization of free Gα subunits at intracellular organelles (49). It should also be noted that we did not block protein synthesis in our experiments, therefore some of the effects we observed could have been due to newly synthesized proteins, particularly with overnight expression of mislocalized DHHC enzymes. However, mislocalization of newly synthesized Gα after 2BP treatment or DHHC-KASH expression would lead us to overestimate the importance of acyl cycling for plasma membrane localization of mature heterotrimers and thus strengthen our main conclusion.

In summary, while a deacylation–reacylation cycle helps maintain the steady-state subcellular distribution of monomeric G proteins, an acylation cycle is far less important for heterotrimeric G proteins. At least in HEK 293 cells, depalmitoylation does not allow a large population of endogenous heterotrimers to sample intracellular compartments through the cytosol.

## Experimental procedures

### Cell culture and transfection

Human embryonic kidney HEK 293 cells (ATCC; CRL-1573) were propagated in 100 mm dishes, on 6-well plates, or on 25 mm round coverslips in high glucose DMEM (Cytiva) and 10% fetal bovine serum (Cytiva) supplemented with penicillin, streptomycin and glutamine (Gibco). Cells were transfected in growth medium using linear polyethyleneimine MAX (Polysciences) at a nitrogen/phosphate ratio of 20 and were used for experiments ∼24 h later. Up to 3.0 μg of plasmid DNA was transfected into each well of a 6-well plate.

### Gene editing

Ribonucleoprotein (RNP) complexes were assembled *in vitro* in IDT nuclease-free duplex buffer from Alt-R crRNA, Alt-R tracrRNA and Alt-R S.p. Cas9 Nuclease V3 purchased from Integrated DNA Technologies (IDT). RNP and repair ssODNs (dissolved in nuclease-free water) were added to single-cell suspensions (10 μl of 1.2 × 10^4^ cells μl^−1^) and electroporated using a Neon Transfection device (Invitrogen) following the manufacturer’s instructions. Cells were expanded and diluted into 48-well plates and grown for 3 weeks. Wells containing single-cell colonies were duplicated into 12-well plates and screened for HiBit expression by mixing crude lysates with purified LgBit protein (Promega) and measuring luminescence in the presence of 5 μM furimazine. After clone expansion, genomic DNA was extracted using the GeneJET Genomic DNA purification kit (ThermoFisher) and used as a template for amplicon sequencing. Sequencing primers were designed to span the editing site and to produce amplicons less than 500 base pairs in length. Amplicon sequencing was performed by Azenta Life Sciences (Amplicon-EZ) and analyzed using CRISPResso2. Cell lines used for experiments had correctly edited alleles but were hemizygous due to competing repair mechanisms. Guide RNAs and homology-directed repair (HDR) templates for mNG-β1 and HiBit-β1 cells were described previously (27).

The human *HRAS* gene was targeted at a site corresponding to the N-terminus (after residue 2) of the HRas protein; the guide sequence 5′-GATGACGGAATATAAGCTGG-3′ was incorporated into the crRNA. The ssODN HDR template sequence for HiBit-HRas cells was: TGGCAGGTGGGGCAGGAGACCCTGTAGGAGGACCCCGGGCCGCAGGCCCCTGAGGAGCGATGACG*GGTGGATCT***GTGAGCGGCTGGCGGCTGTTCAAGAAGATTAGC***GGCGGAAGCGGT*GAATATAAGCTGGTGGTGGTGGGCGCCGGCGGTGTGGGCAAGAGTGCGCTGACCATCCAG, with the HiBit tag sequence in **bold**, and GGSG and GGS linkers in *italic*s.

The human *GNG5* gene was targeted at a site corresponding to the N-terminus (after residue 1) of the Gγ5 protein; the guide sequence 5′-GACGCTGGAGGAGCCAGACATGG-3′ was incorporated into the crRNA. The ssODN HDR template sequence for HiBit-γ5 cells was: TCTGGCCCCGCCGACCCACGGCCCACGACCCACCGACCCACGAATCGGCCCGGCCGTCGCGTGCACCATG**GTGAGCGGCTGGCGGCTGTTCAAGAAGATTAGC***GGTGGATCTGGCGGAAGCGGT*TCTGGCTCCTCCAGCGTCGCCGCTATGAAGAAAGTGGTTCAACAGCTCCGGCTGGAGGCCGGACTCAACCG, with the HiBit tag sequence in **bold**, and GGSGGSG linker in *italic*s.

The human *GNG12* gene was targeted at a site corresponding to the N-terminus (after residue 2) of the Gγ12 protein; the guide sequence 5′-GTTGGTGCTTGCTGTTTTGCTGG-3′ was incorporated into the crRNA. The ssODN HDR template sequence for HiBit-γ12 cells was: GATGCCTGTTACTTGTCTTTAAAAAAAATTCTCCTCAGATTTCAGGTAAAAACAATAATTGAAGATGTCC**GTGAGCGGCTGGCGGCTGTTCAAGAAGATTAGC***GGTGGATCTGGCGGAAGCGGT*AGCAAAACAGCAAGCACCAACAATATAGCCCAGGCAAGGAGAACTGTGCAGCAGTTAAGATTAGAAGCCTCCAT, with the HiBit tag sequence in **bold**, and GGSGGSG linker in *italics*.

### Plasmids

DHHC/S-KASH, mRuby2-PTP1b, CMV-LgBit, Venus-Kras, Venus-PTP1b, mem-link-Venus, memGRKct-Venus were described previously (27, 29, 32, 50). Venus-Ras binding domain (Venus-RBD) was constructed by ligating a synthetic fragment encoding the Ras binding domain of Raf1 with a GGSG linker into pVenus-C1 using *XhoI* and *BamHI*. ER-localized Gα1i (ER-Gi1) was made by replacing the first 6 amino acids of Gαi1 with an ER-targeting sequence from CYP2C1 (MDPVVVLGLCLSCLLLLSLWKQSY) and a GGG linker by ligating a synthetic fragment after digestion with *HindIII* and *BsrGI*. Venus-KASH was made by replacing mRFP in mRFP-KASH (32) with Venus from pVenus-C1 using *NheI* and *BsrGI*. SNAPf-β_2_AR, SNAPf-D2R and SNAPf-M3R were kindly provided by Jonathan Javitch (Columbia University). All plasmids were verified by automated sequencing (Plasmidsaurus).

### Imaging

Imaging of mNG-β1 cells was performed on a Leica SP8 laser scanning confocal microscope using a 63 × 1.40 NA oil immersion objective. Cells grown on 25 mm round coverslips were transferred to a steel imaging chamber and imaged in HEPES Imaging (HI) buffer which contained 150 mM NaCl, 10 mM NaHEPES, 5 mM glucose, 2.5 mM KCl, 1.2 mM CaCl_2_, 1 mM MgCl_2_ (pH 7.2). For the experiments shown in Figure 1, cells were transfected with 0.1 μg mRuby2-PTP1b with or without 0.5 μg of ER-Gαi1 per coverslip; mNG-β1 was excited at 488 nm and detected at 495 to 545 nm, and mRuby2-PTP1b was excited at 552 nm and detected at 565 to 665 nm. All image analysis was carried out using ImageJ and raw images. To measure mNG-β1 fluorescence localized to the ER, a mask was created by thresholding the mRuby2-PTP1b channel. For the construction of figures images were exported as .TIF files with or without uniform contrast enhancement applied by ImageJ.

### BRET

For bystander BRET monitoring of G protein localization, cells were transfected in 6-well plates with 1 μg per well of a Venus-tagged compartment marker and 0.1 μg per well of CMV-LgBit. 2-bromopalmitate (2BP; final concentration 50 μM) or DMSO were added directly to the growth medium for the indicated time. For measurements, cells were resuspended in Dulbecco’s phosphate-buffered saline (DPBS). For long-term agonist stimulation HiBit-β1, HiBit-γ5, HiBit-γ12, cells expressing CMV-LgBit (0.1 μg per well) and either SNAPf-β2AR, SNAPf-D2R or SNAPf-M3R (0.1 μg per well) were incubated with agonist for 6 h in the incubator, then washed and resuspended in DPBS containing antagonist prior to reading BRET. Agonists were isoproterenol (10 μM), dopamine (100 μM) and acetylcholine (100 μM); antagonists were ICI 118551 (10 μM), haloperidol (10 μM) and atropine (10 μM); all small-molecule ligands were obtained from Millipore Sigma or Cayman Chemical. For functional validation of HiBit-β1 cells, muscarinic M4R (0.5 μg per well), CMV-LgBit (0.1 μg per well) and memGRK3ct-Venus (0.5 μg per well) were transfected. Cells were resuspended in DPBS, and kinetic BRET measurements were made during sequential injection of acetylcholine and atropine at the concentrations listed above. For functional validation of HiBit-HRas cells, CMV-LgBit (0.1 μg per well) and Venus-RBD (0.5 μg per well) were transfected. Cells were serum starved for 5 h, resuspended in DPBS, and kinetic BRET measurements were made during sequential injection of epidermal growth factor (EGF; 100 ng ml^−1^) and gefitinib (1 μM). All BRET measurements were made in buffer solutions containing the substrate furimazine (Promega or ChemShuttle; 1:1000 from a 5 mM stock dissolved in 90% ethanol/10% glycerol). Steady-state BRET measurements were made using a Mithras LB940 photon-counting plate reader (Berthold Technologies GmbH) or Tecan Spark photon-counting plate reader (Tecan Austria GmbH). Kinetic BRET measurements were made using a Lumistar Optima plate reader (BMG Labtech). Raw BRET signals were calculated as the emission intensity at 520 to 545 nm divided by the emission intensity at 475 to 495 nm. Net BRET signals were calculated as the raw BRET signal minus the raw BRET signal measured from cells expressing only the donor.

### SDS-PAGE

Pelleted cells were mixed with Laemmli buffer (Bio-Rad), and proteins were separated on four to 15% SDS polyacrylamide gradient gels (Bio-Rad), then transferred to polyvinylidene difluoride (PVDF) membranes (Millipore Sigma). HiBit-tagged proteins were detected using the NanoGlo HiBit Blotting kit (Promega) following the manufacturer’s instructions, and membranes were imaged using an Amersham Imager 600.

### Statistical analysis

All statistical testing was carried out using GraphPad Prism version 10.4.0. Exact *p* values are given in the figures or Supporting Information Source Data File.

### Data availability

All study data are included in the article and are provided in the Source Data File. Cell lines and plasmids generated for this study are freely available upon request from the corresponding author. No unique code or software was used for the study.

## Supporting information

This article contains supporting information.

## Conflict of interest

The authors declare that they do not have any conflicts of interest with the content of this article.

## Supporting information

Supporting Information

Acyl cycling source data and P values
