# Supplementary material for: The role of acyl cycling in endogenous G protein localization
Source: J Biol Chem. 2025 Dec 12;302(2):111045. doi: 10.1016/j.jbc.2025.111045 (PMC12804145; doi:10.1016/j.jbc.2025.111045)
Supplement: Supporting Information [file mmc1.pdf]

Supporting Information

The role of acyl cycling in endogenous G protein localization

Wonjo Jang[1], Kanishka Senarath[1], Sumin Lu[1], Gonzalo P. Solis[2], Vladimir L. Katanaev[2,3] and Nevin A. Lambert[1]

[1]Department of Pharmacology and Toxicology, Medical College of Georgia, Augusta University, Augusta, GA, USA
[2]Translational Research Center in Oncohaematology, Department of Cell Physiology and Metabolism, Faculty of Medicine, University of Geneva, Geneva, Switzerland
[3]Translational Oncology Research Center, Qatar Biomedical Research Institute, Hamad Bin Khalifa University, Doha, Qatar

Correspondence: nelambert@augusta.edu
ORCID: 0000-0001-7550-0921

Figure S1

Figure S2

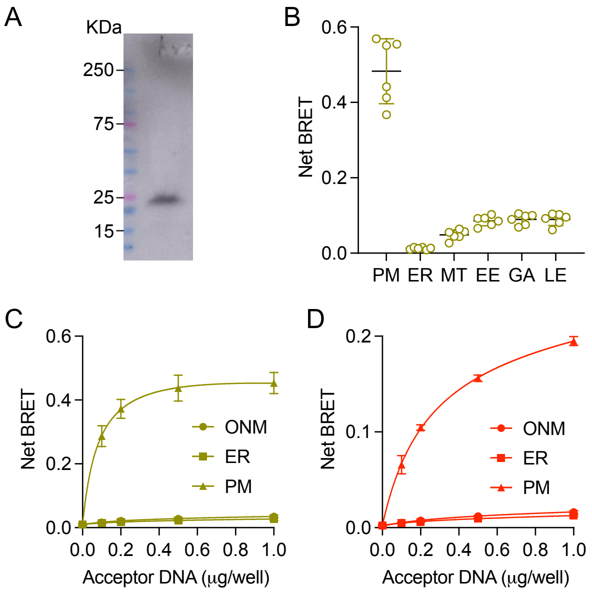

Figure S1. **Characterization of HiBit-HRas cells.** *A*, SDS-PAGE analysis of HiBit-HRas cell lysates; the predicted molecular weight of the edited gene product is 23.06 kilodaltons (KDa); representative of 2 independent experiments. *B*, bystander net BRET signals between HiBit-HRas and Venus-tagged markers of the plasma membrane (PM), endoplasmic reticulum (ER), mitochondria (MT), early endosomes (EE), Golgi apparatus (GA) and late endosomes (LE); mean ± S.D.; *n*=6 independent experiments. *C* and *D*, bystander net BRET between HiBit-HRas (*C*) or HiBit-β1 (*D*) and Venus-tagged markers of the plasma membrane (PM), endoplasmic reticulum (ER) and outer nuclear membrane (ONM) as a function of marker expression; mean ± S.D.; *n*=4-5 independent experiments.

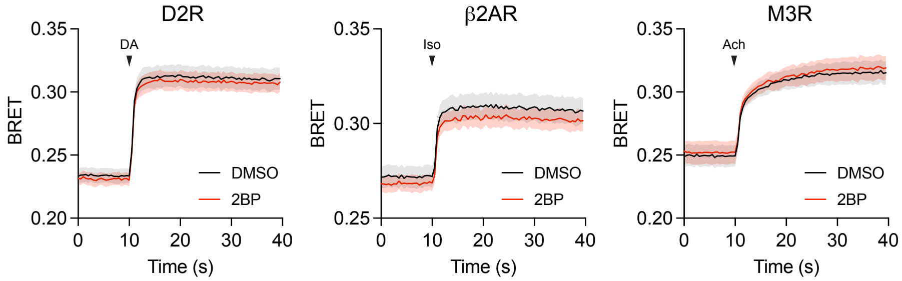

Figure S2. **Treatment with 2BP does not inhibit G protein activation.** HiBit-β1 cells expressing dopamine D2 receptors (D2R), β2 adrenoreceptors (β2AR), or M3 muscarinic acetylcholine receptors (M3R) and the free Gβγ sensor GRKct-Venus were treated with vehicle (DMSO) or 2BP (50 μM) for 2 hours then stimulated with dopamine (DA; 100 μM), isoproterenol (Iso; 10 μM) or acetylcholine (Ach; 100 μM) respectively. Traces represent the mean ± S.E.M. of 12 replicates from 3 independent experiments.
